# Two-Step Estimation of the Impact of Contextual Variables on Technical Efficiency of Hospitals: The Case Study of Public Hospitals in Iran

**DOI:** 10.3389/fpubh.2021.785489

**Published:** 2022-01-06

**Authors:** Efat Mohamadi, Mohammad Mehdi Kiani, Alireza Olyaeemanesh, Amirhossein Takian, Reza Majdzadeh, Farhad Hosseinzadeh Lotfi, Hamid Sharafi, Haniye Sadat Sajadi, Zahra Goodarzi, Somayeh Noori Hekmat

**Affiliations:** ^1^Health Equity Research Center (HERC), Tehran University of Medical Sciences (TUMS), Tehran, Iran; ^2^Department of Health Management and Economics, School of Public Health, Tehran University of Medical Sciences (TUMS), Tehran, Iran; ^3^National Institute of Health Research, Tehran University of Medical Sciences (TUMS), Tehran, Iran; ^4^Department of Global Health and Public Policy, School of Public Health, Tehran University of Medical Sciences (TUMS), Tehran, Iran; ^5^Community Based Participatory Research Centre and Knowledge Utilization Research Center, Tehran University of Medical Sciences (TUMS), Tehran, Iran; ^6^Department of Mathematics, Science and Research Branch, Islamic Azad University, Tehran, Iran; ^7^Knowledge Utilization Research Center, Tehran University of Medical Sciences, Tehran, Iran; ^8^University Research and Development Center, Tehran University of Medical Sciences, Tehran, Iran; ^9^Research Center for Health Services Management, Institute for Futures Studies in Health, Kerman University of Medical Sciences, Kerman, Iran

**Keywords:** contextual factors, technical efficiency, DEA, public hospitals, inefficiency, health policy

## Abstract

**Background:** Measuring the efficiency and productivity of hospitals is a key tool to cost contamination and management that is very important for any healthcare system for having an efficient system.

**Objective:** The purpose of this study is to examine the effects of contextual factors on hospital efficiency in Iranian public hospitals.

**Methods:** This was a quantitative and descriptive-analytical study conducted in two steps. First, we measured the efficiency score of teaching and non-teaching hospitals by using the Data Envelopment Analysis (DEA) method. Second, the relationship between efficiency score and contextual factors was analyzed. We used median statistics (first and third quarters) to describe the concentration and distribution of each variable in teaching and non-teaching hospitals, then the Wilcoxon test was used to compare them. The Spearman test was used to evaluate the correlation between the efficiency of hospitals and contextual variables (province area, province population, population density, and the number of beds per hospital).

**Results:** On average, the efficiency score in non-teaching hospitals in 31 provinces was 0.67 and for teaching hospitals was 0.54. Results showed that there is no significant relationship between the efficiency score and the number of hospitals in the provinces (*p* = 0.1 and 0.15, respectively). The relationship between the number of hospitals and the population of the province was significant and positive. Also, there was a positive relationship between the number of beds and the area of the province in both types of teaching and non-teaching hospitals.

**Conclusion:** Multilateral factors influence the efficiency of hospitals and to address hospital inefficiency multi-intervention packages focusing on the hospital and its context should be developed. It is necessary to pay attention to contextual factors and organizational architecture to improve efficiency.

## Introduction

Health promotion and response to the demands of people and society is the main mission of the health system (1). In this regard, hospitals, as the main facilities for health services, have a special role in the health system (2). Further, a hospital is a very complex social organization that plays a significant role in the maintenance and promotion of health but provides such complex and specialized services which are also expensive (3).

Hospital expenditures represent around 30–80% of the total health expenditures in all high and low-middle income countries (4). Therefore, assessing the efficiency and productivity of hospitals is a key tool to cost contamination and management that is important for any healthcare system due to having an efficient system. That is why one of the middle goals of the health systems is the improvement of efficiency (5, 6). Because of that, health policymakers measure the efficiency of hospitals to achieve an efficient health system.

There are many studies that have been conducted on the efficiency measurement using the Data Envelopment Analysis (DEA) approach in different contexts and countries, for example, studies of Ersoy et al. (7), which were among the first efforts in the field of efficiency analysis using the DEA technique. Kirigia et al. (8), Ramanathan (9), Ghaderi et al. (10), Mohammadi et al. (11), and Azad et al. (12) have used the frontier data analysis method to evaluate the efficiency of hospitals. Some studies have used the DEA method by applying and promoting this method. For example, in the study of Manh-TrungPhung et al., applying a new DEA modeling technique to be demonstrated in managerial implications can improve the efficiency of the system (13). Another study proposes the Multi-Objective Programming (MOP) method for solving network DEA (NDEA) models. Kao et al. (14) apply the idea of cross-evaluation, which has been demonstrated to be an effective approach in ranking Decision Making Units (DMUs) for systems considered as a whole-unit to measure the efficiency of the two basic structures of network systems, namely, series and parallel (15). Also, Kao et al. propose a general slack-based measure (SBM) model for network systems and can decompose the system efficiency into a weighted average of the process efficiencies (16).

There are abounding variables that affect hospital efficiency. These variables are considered input and output variables to measure efficiency. Nevertheless, the impact of contextual factors (i.e., the population covered by the hospital, type of hospitals, management of hospitals, and qualitative variables) on hospital efficiency as external determinants are rarely paid attention in efficiency measurement. In contrast to most previous studies that have focused primarily on examining the impacts of inputs (number of physicians, nurses, money, etc.) on technical efficiency, this study also investigates the effect of contextual and environmental factors on hospital efficiency.

We conducted this study on the Iran health system. Iran is an ancient country located in the Middle East, a region between Asia, Europe, and Africa. The area of Iran is 1,648,195 km^2^, which makes it the 17th largest country in the world. Iran is divided into 31 provinces and 336 districts. The Ministry of Health and Medical Education (MOHME) is the stewardship of heath in Iran. All hospitals are regulated under the supervision of the MOHME. There are 921 active hospitals in the country, 80% of them are public (Governmental) and 20% are non-public hospitals. Governmental hospitals are divided into three categories: Medical-Non-Teaching hospitals, Medical-Teaching hospitals, and Medical-Teaching-Research hospitals.

In this study, we assume that contextual factors have an important role in hospitals that can affect hospital efficiency. The idea that contextual or environmental factors have a significant role in explaining the deviation of actual values from the frontier, has long been an underlying motivation in frontier models in production economics. The results of this study lead to a better understanding of the relationship between contextual factors. In addition, determining their interactions can help efficient policymaking to improve hospital efficiency. Therefore, the purpose of this study is to examine the effects of contextual factors on hospital efficiency in Iranian public hospitals.

## Methods

This was a quantitative and descriptive-analytical study that was conducted in two steps. First, we measured the efficiency score for teaching and non-teaching hospitals. Second, the relationship between efficiency score and contextual factors was analyzed.

### Data Collection

According to the purpose of the study, we had two categories of data, namely, data related to measuring the efficiency of hospitals and data related to contextual variables. For measuring hospital efficiency, we conducted a qualitative analysis, i.e., literature review and collecting opinions of experts to identify the input and output indicators. First, a scoping review of related studies identified a list of related indicators for the objectives of our research (17). Second, we examined the existence of data associated with each indicator and the reliability of the data source, according to which, many indicators were excluded. Finally, the included indicators were reviewed and approved by an expert panel, comprising of the research team plus selected key informants in the field of health management, policy, and economics.

We considered data of all the Iranian Medical and Non-teaching and Medical and teaching hospitals affiliated with MOHME. There were 577 active hospitals in these categories that were scattered across 31 provinces in Iran. We extracted data from secondary databases linked to the health information system (HIS) of MOHME in 2016. We used a checklist for data collection that was designed based on the input and output variables. An Excel sheet was used to enter the data as “Decision Making Units (DMUs)” for all teaching and non-teaching public hospitals. We then cleaned up the data to ensure the existence and accuracy of all data for each indicator per DMU. Irregular data was compared with other sources to ensure data integrity. Data collection and cleaning lasted 6 months.

For analyzing the impact of contextual factors affecting hospital efficiency, we determined some contextual variables: number of hospitals in the province, area of the province, population of the province, population density, and number of beds per hospital by type of hospital. The data source of this step included the HIS and Iran Statistics Center Database. Data was gathered for 2016.

### Data Analysis

We considered each hospital as a DMU while hospitals were categorized into various specialty groups and Extended Data Envelopment Analysis (EDEA) models were independently implemented to each categorization.

We considered 4 input and 7 output for measuring the efficiency of hospitals. We use the following symbols to show the values of inputs and outputs of the hospital j (j = 1…, n).


$$\begin{array}{l} {\text{x}}_{\text{ij}}:\text{Value of ith input of hospital j},\;\text{ i}=\text{1},\;..,\text{4},\text{ j}=\text{1},\;...,\text{ n}.\\ {\text{y}}_{\text{rj}}:\text{Value of rth output of hospital j},\;\text{ r}=\text{1},\;...,\text{7},\text{ j=1},\;...,\text{ n}. \end{array}$$

As described above, we determined the inputs and outputs for each hospital for modeling as Table 1:

**Table 1:** Inputs and outputs for each hospital.

**Inputs**	**Symbols**	**Outputs**	**Symbols**
n. Physician	*x*_1*j*_	n. Inpatient	(*D*)*y*_1*j*_
n. Nurse	*x*_2*j*_	n. Outpatient	(*D*)*y*_2*j*_
n. Other staff	*x*_3*j*_	n. Surgical operation	(*D*)*y*_3*j*_
n. Hospital bed	*x*_4*j*_	Degree of accreditation	(*D*)*y*_4*j*_
		Average length of stay	(*U*.*D*)*y*_5*j*_
		Number of bed days	(*D*)*y*_6*j*_
		Bed turnover	(*D*)*y*_7*j*_

Symbols D and U.D are desirable and undesirable, respectively. In other words, an increase of desirable outputs is considered by management which improves productivity. However, the undesirable outputs are not considered by the manager which has an adverse effect on productivity. Since the fifth output (Average length of stay) is undesirable. We make the following changes to make it a desirable output.


(1) $$\begin{array}{l} {\text{y}}_{5{\text{j}}_{\text{new}}}=1/{\text{y}}_{5{\text{j}}_{\text{previous}}} \end{array}$$

As we mentioned in the method, given the definitions of each input and output, the following constraints are taken for them based on the opinions of experts.


$$\begin{array}{r} \begin{array}{ccc} v_{1}\geq 1.3v_{2}, & u_{7}\geq u_{1}, & u_{6}\geq u_{3},\\ v_{2}\geq 1.3v_{3}, & u_{2}\geq u_{1}, & u_{3}\geq 1.5u_{4}\\ v_{2}\geq 3.9v_{4}, & u_{3}\geq u_{5}, \end{array} \end{array}$$

Relationships (2) show the relative weight of indicators. For example, the importance of the seventh output is at least equal to the first output, and the importance of the first input to the second input is at least 1.3. Since the design of this research requires a restriction, the modeling is done in envelopment form. Therefore, constraints (2) appear in a trade-off in the envelopment form with symbols α and β. Also, the variables γ and μ correspond to this trade-off in envelopment form.

On the other hand, the sixth output is expressed as a “percentage,” so its value must always be between [0, 100]. Therefore, the following constraints are considered in the modeling.


(2) $$\begin{array}{r} 0\leq \overset{315}{\underset{j=1}{\sum }}\lambda_{j}y_{6j}+\overset{5}{\underset{j=1}{\sum }}\gamma_{j}\beta_{6j}\leq 100 \end{array}$$

The number of bed days is also dependent on the number of beds, which is why the following model constraints are considered in the modeling.


(3) $$\begin{array}{r} \overset{315}{\underset{j=1}{\sum }}\lambda_{j}y_{7j}+\overset{5}{\underset{j=1}{\sum }}\gamma_{j}\beta_{7j}\leq 365*\left(\overset{315}{\underset{j=1}{\sum }}\lambda_{j}x_{4j}+\overset{3}{\underset{j=1}{\sum }}\mu_{j}\alpha_{4j}\right) \end{array}$$

According to the above description, the radial model in the envelopment form, taking into according to the trade-off and limitations on the template, will be as follows:

The final model to calculate the relative efficiency of hospital p can be found by solving the model hereunder:


$$\begin{array}{c} \begin{array}{c} Min\;\theta \\ s.t. \end{array} \end{array}$$

(a) $$\begin{array}{c} \overset{n}{\underset{j=1}{\sum }}\lambda_{j}x_{1j}+\overset{3}{\underset{j=1}{\sum }}\mu_{j}\alpha_{4j}\leq \theta x_{ip},\;i=1,...,4, \end{array}$$

(b) $$\begin{array}{c} \overset{n}{\underset{j=1}{\sum }}\lambda_{j}y_{rj}+\overset{5}{\underset{j=1}{\sum }}\gamma_{j}\beta_{rj}\geq y_{rp},\;r=1,...,7, \end{array}$$

(c) $$\begin{array}{c} \overset{n}{\underset{j=1}{\sum }}\lambda_{j}y_{6j}+\overset{5}{\underset{j=1}{\sum }}\gamma_{j}\beta_{6j}\leq 100, \end{array}$$

(d) $$\begin{array}{c} \overset{n}{\underset{j=1}{\sum }}\lambda_{j}y_{6j}+\overset{5}{\underset{j=1}{\sum }}\gamma_{j}\beta_{6j}\geq 0, \end{array}$$

(e) $$\begin{array}{c} \overset{n}{\underset{j=1}{\sum }}\lambda_{j}y_{7j}+\overset{5}{\underset{j=1}{\sum }}\gamma_{j}\beta_{7j}\leq 365*\left(\overset{n}{\underset{j=1}{\sum }}\lambda_{j}x_{4j}+\overset{3}{\underset{j=1}{\sum }}\mu_{j}\alpha_{4j}\right) \end{array}$$

(5) $$\begin{array}{c} \lambda_{j}\geq 0,\;j=1,...,n,\\ \gamma_{j}\geq 0,\;j=1,...,5,\\ \mu_{j}\geq 0,\;j=1,2,3. \end{array}$$

The optimal value of the objective function of the model (5) can be denoted as a relative efficiency of hospital p. It is obvious that if the optimal value of the objective function of the model (3) is equivalent to 1, then hospital p is efficient. Similarly, if the optimal value of the objective function of the model (3) is <1, then the hospital p can be called as being inefficient. Data were analyzed through GAMS software 24.3.

In the second step of the study, we used median statistics (first and third quarters) to describe the concentration and distribution of each variable in teaching and non-teaching hospitals. Then, the Wilcoxon test was used to compare them. The Spearman test was used to evaluate the correlation between the efficiency of hospitals and contextual variables (province area, province population, population density, and the number of beds per hospital in 31 provinces). In addition, the spline smoothing (with strong regression) was added in the data distribution chart to give a better description of the relationship between the variables (especially in outlier data). In all tests, a significance level of 0.05 was considered. Data were analyzed using R software 4.0.2.

## Results

The descriptive statistics of inputs, outputs, and explanatory variables are shown in Tables 2, 3. We summarized the efficiency score of hospitals in Table 4. Tables 5, 6 show the efficiency score of the general and specialized hospitals during 2012–2016 in Iran.

**Table 2:** Descriptive statistics of inputs and Output variables to measure efficiency.

**Variable**	**Medical and non-teaching**		**Medical and teaching**
**Input variables**			
n. Physician	Mean ± Sd	30 ± 21	67 ± 51
	(Min; Max)	(2; 116)	(4; 361)
n. Nurse	Mean ± Sd	121 ± 81	330 ± 208
	(Min; Max)	(8; 614)	(14; 1464)
n. Other staff	Mean ± Sd	130 ± 66	320 ± 249
	(Min; Max)	(13; 412)	(43; 2227)
n. Hospital bed	Mean ± Sd	88 ± 55	242 ± 149
	(Min; Max)	(3; 335)	(15; 828)
**Output variables**			
n. Inpatient	Mean ± Sd	8,522 ± 5,884	24,181 ± 14,504
	(Min; Max)	(93; 40,685)	(102; 80,867)
n. Outpatient	Mean ± Sd	1,016,575 ± 940,923	1,414,848 ± 1,553,272
	(Min; Max)	(400; 4,690,700)	(4,787;6,967,190)
n. Surgical operation	Mean ± Sd	3,427 ± 2,522	10,074 ± 7,480
	(Min; Max)	(50; 14,072)	(30; 44,318)
Average length of sta	Mean ± Sd	2.3 ± 0.8	3 ± 1
	(Min; Max)	(1; 10)	(1.1; 6.6)
Bed occupation (%)	Mean ± Sd	62 ± 22	77 ± 15
	(Min; Max)	(5; 100)	(4.7; 108)
Number of bed days	Mean ± Sd	22,817 ± 19 485	74,742 ± 53,973
	(Min; Max)	(130; 104,523)	(200; 342,302)
Accreditation level	Mean ± Sd	2.4 ± 0.6	2.9 ± 0.4
	(Min; Max)	(1; 4)	(2; 4)

**Table 3:** Descriptive statistics and comparison of variables between educational and non-educational hospitals.

**Characteristic**		Median (Min; Max)^*^	*p*-value^**^
Hospitals in a province	Medical and non-teaching	6 (4, 8)	0.019
	Medical and teaching	10 (5, 16)	
Number of beds per hospitals in a province	Medical and non-teaching	205 (167, 248)	<0.001
	Medical and teaching	87 (74, 99)	
Province area		28,294 (18,584, 72,087)	–
Province population		1,760,649 (1,150,966, 3,214,968)	–
Population density of provinces		55 (35, 86)	–

* *Median statistics (first and third quarters)*.

** *Wilcoxon test*.

**Table 4:** Overall efficiency results.

**Summary statistics**	**Medical and non-teaching**	**Medical and teaching**
*N*	343	234
Median	0.671	0.543
Mean	0.572	0.694
Max	1	1
Min	0.423	0.566
Efficient (N) (%) (X > 0.8)	7 (22%)	5 (16%)
Inefficient (N) (%) (X < 0.8)	21 (77%)	26 (84%)

**Table 5:** The relationship between efficiency score and contextual variables.

** 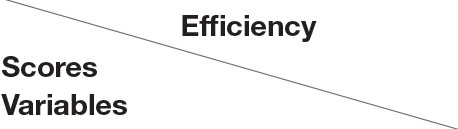 **		**Efficiency of medical and non-teaching**	**Efficiency of medical and teaching**
Number of hospitals	Lower	−0.44	0.21
	*R*	−0.10	0.15
	Upper	0.25	0.48
	*P*	0.56	0.42
Number of beds per hospitals	Lower	0.14	0.08
	*R*	0.53	0.49
	Upper	0.77	0.76
	*P*	0.01	0.02
Population density	Lower	0.07	0.23
	*R*	0.49	0.6
	Upper	0.76	0.82
	*P*	0.02	0.003

**Table 6:** The relationship between number of hospitals/number of beds with contextual factors.

**Variables**		**Area of the province**		**Population of the provinces**		**Population density**	
		**Non-teaching**	**Teaching**	**Non-teaching**	**Teaching**	**Non-teaching**	**Teaching**
Number of hospital	Lower	0.1018	0.7218	0.5457	0.7218	0.1727	0.05374
	*R*	0.7542	0.8588	0.7542	0.8588	0.1934	0.3126
	Upper	0.8748	0.931	0.8748	0.931	0.5126	0.6048
	*P*	<0.001	<0.001	<0.001	<0.001	0.2971	0.09261
Number of beds	Lower	0.08096	0.0589	0.7006	0.6328	0.05106	0.1204
	*R*	0.2815	0.4105	0.8452	0.8087	0.3089	0.2507
	Upper	0.5781	0.6714	0.9231	0.9052	0.5978	0.5604
	*P*	0.1251	0.02426	<0.001	<0.001	0.0909	0.1814

Based on findings, the mean of beds in Medical and Non- teaching hospitals is 88 (SD = 55), which is lower than beds in Medical and teaching hospitals (242 ± 149). The input and output variables in Medical and teaching hospitals are higher than in Medical and Non-teaching hospitals (Table 2).

According to the data collected from 31 provinces, the average number of beds in non-teaching hospitals was higher than in teaching hospitals. However, this difference was not statistically significant (*p* = 0.083). Also, 75% of the provinces have between 4 and 8 Medical and Non-teaching hospitals and 5–16 Medical and teaching hospitals (Table 3).

### Efficiency Score

On average, the efficiency score in non-teaching hospitals in 31 provinces was 0.67, and for teaching hospitals was 0.54. Results showed that the efficiency of non-teaching hospitals is significantly higher than that of teaching hospitals. In addition, it can be said that 75% of the non-teaching hospitals in the provinces have an efficiency score between 0.64 and 0.70. While the efficiency score for 75% of teaching hospitals is between 0.51 and 0.63 (Table 3).

### The Relationship Between Efficiency Score and Contextual Variables

According to the findings, there is no significant relationship between the efficiency score and the number of hospitals in any of the teaching and non-teaching hospitals in the provinces (*p* = 0.1 and 0.15, respectively). But it can be said that there is a significant relationship between efficiency score and bed number between provinces (*p* = 0.01, *p* = 0.02). Also, there is a significant relationship between efficiency score and population density of provinces (*p* = 0.02, *p* = 0.003) (Table 5).

According to the findings, the relationship between the number of hospitals and the population of the province is significant and positive. Also, generally, it can be said that the provinces with higher populations have more hospitals. According to the results, it can be said that the relationship between the number of hospitals and the population density of the province is positive. In addition, the intensity of the relationship between the number of hospitals and population density is higher for teaching hospitals (Table 6).

According to Spearman's correlation test, the relationship between the number of beds and the area of the province in both types of teaching and non-teaching hospitals is positive. Although this relationship is not significant in Non-teaching hospitals (*p* = 0.125), it is significant in teaching hospitals (Table 6).

According to Spearman's correlation test, the relationship between the number of beds and the population of the province is positive and significant. These results can be seen in the distribution charts. With these results, it cannot be claimed that population growth has increased the number of beds, but simply states that most provinces with more populations have more beds (Table 6).

Given the estimated value of the Spearman correlation, it can be said that the relationship between the number of beds and the population density of the province is positive. In addition, the relationship between the number of beds and the population density is higher for teaching hospitals.

## Discussion

The objective of this study was to measure hospital efficiency and to examine the effects of contextual factors on hospital efficiency in Iran at national and sub-national levels. At the macro level, controlling some contextual factors of provinces was beyond the managerial level. To identify managerial inefficiency, which is important to resource allocation, the effects of these factors must be accounted for comparing organizations (18). This is especially true in Iran because there are several different territories with regards to demographic, economic, social, and environmental aspects.

The first result of this study showed that the median score of efficiency was 0.67 and 0.54 in non-teaching hospitals and teaching hospitals, respectively. The result showed that the efficiency of non-teaching hospitals is significantly higher than teaching hospitals. Whether a hospital is specialized or general or teaching or non-teaching plays an important role in the economic performance of them to create different motivations for economic practice (19). Therefore, these variables have been a noteworthy interest of researchers for a long time, and several types of research have been studied on these issues (20–23).

A recent study calculated and analyzed the efficiency of all public hospitals in Spain in 2017 (24) and reported that the average efficiency score was 0.736. The study compared similar hospitals with each other. In our study, we used more output variables to enhance the reliability of the analysis. A systematic review showed that 90% of studies used the DEA method to measure the efficiency of hospitals in Iran, and the calculated score ranged from 0.7 to 0.9 (25).

A similar study in China also used the DEA method (26). They used the number of beds as the input variable and the hospitalization days, the number of visits, and the number of surgical operations as the most-used output variables for measuring efficiency. A Chinese study measured the efficiency of government hospitals to examine the impact of the country-wide development plan of 2009 on the efficiency of a sample of 114 hospitals. They used similar input and output variables to our study and calculated the average efficiency score of 0.748, while the significant potential for improving the technical efficiency of the hospitals was reported (27).

Another study used similar input and output indicators to examine the efficiency of health services centers in Indonesia. They used Pabón-Lasso model. Forty percent of hospitals and 33% of health centers were located in the high-performing sector of the Pabón-Lasso model (28).

Another study used 10 variables to measure the efficiency of Turkish hospitals in 2015 and found that only 17% of the total 1,103 hospitals were efficient (29). A similar study in Turkey that examined the efficiency of 1,079 hospitals reported that the government hospitals affiliated with the Turkish Ministry of Health were more efficient than the private hospitals (30).

There is no significant relationship between the efficiency score and the number of hospitals in any of the teaching and non-teaching hospitals. Teaching activities are an important cost-driving factor and hospitals that have a broader range of specialization are relatively more costly (31).

Despite this, there was no significant relationship between the efficiency score and the number of hospitals in any of the teaching and medical hospitals while there was a significant relationship between efficiency score and number of beds. Some studies reported consistent evidence of economies of scale for hospitals with 200–300 beds. Inefficiency can be expected to occur more in hospitals with below 200 beds and above 600 beds. Nevertheless, economies of scale depended upon the category of the hospitals in addition to the number of beds and volume of output (25, 32–34). Another study showed that more than three and <5 beds per 1,000 population significantly influenced the efficiency score (35). Also, the results of another study showed that the size, type, and ownership of hospitals had an effect on the degree of their technical efficiency (36). Policymakers and hospital managers should consider the appropriate number of beds for the hospital as it prevents wastage of resources. It seems that the reason for the inefficiency of most hospitals is the lack of adequate allocation of beds according to geographical conditions. Choosing the optimal amount of beds for a hospital is the first step to improving the efficiency and productivity of hospitals.

Results showed that there was a significant relationship between efficiency score, population density, and the overall population of the province. Some studies have shown that the density of population and bed density significantly influenced the efficiency score (35). Also, the relationship between the number of hospitals and the population density of the province was positive.

The relationship between the number of beds and the area of the province in both types of teaching and non-teaching hospitals was positive. It seems the area of the province and population density determine the location for making a hospital and it can increase the input and resources of the hospital such as patient, money, and another resource improving the efficiency of hospitals.

## Conclusions

There is no one unique intervention that can be adopted by different hospitals to improve hospital efficiency. Multiple factors influence the efficiency of hospitals. To address hospital inefficiency, multi-intervention packages focusing on the hospital and its context should be developed. It is necessary to pay attention to the contextual factors and organizational architecture before any cost contamination and efficiency improvement. It is suggested that the comprehensive hospital's efficiency indicators should be determined to more accurately evaluate the hospital's efficiency.

## Data Availability Statement

The original contributions presented in the study are included in the article/supplementary material, further inquiries can be directed to the corresponding authors.

## Author Contributions

AT, AO, and RM conceived the study and designed its method. EM and MK performed the computations and applied the model, with help from FH for revision of the analytical method. EM, HSh, and SN carried out the analytical experiment. AO and AT wrote the manuscript. All authors discussed the results, contributed to the final manuscript, contributed to the development and approved the final manuscript.

## Conflict of Interest

The authors declare that the research was conducted in the absence of any commercial or financial relationships that could be construed as a potential conflict of interest.

## Publisher's Note

All claims expressed in this article are solely those of the authors and do not necessarily represent those of their affiliated organizations, or those of the publisher, the editors and the reviewers. Any product that may be evaluated in this article, or claim that may be made by its manufacturer, is not guaranteed or endorsed by the publisher.
